# The effect of open lung ventilation on right ventricular and left ventricular function in lung-lavaged pigs

**DOI:** 10.1186/cc4944

**Published:** 2006-06-08

**Authors:** Dinis Reis Miranda, Lennart Klompe, Filippo Cademartiri, Jack J Haitsma, Alessandro Palumbo, Johanna JM Takkenberg, Burkhard Lachmann, Ad JJC Bogers, Diederik Gommers

**Affiliations:** 1 Department of Anesthesiology, Erasmus MC, Rotterdam, The Netherlands; 2 Department of Cardio-Thoracic Surgery, Erasmus MC, Rotterdam, The Netherlands; 3 Department of Radiology, Erasmus MC, Rotterdam, The Netherlands

## Abstract

**Introduction:**

Ventilation according to the open lung concept (OLC) consists of recruitment maneuvers, followed by low tidal volume and high positive end-expiratory pressure, aiming at minimizing atelectasis. The minimization of atelectasis reduces the right ventricular (RV) afterload, but the increased intrathoracic pressures used by OLC ventilation could increase the RV afterload. We hypothesize that when atelectasis is minimized by OLC ventilation, cardiac function is not affected despite the higher mean airway pressure.

**Methods:**

After repeated lung lavage, each pig (*n *= 10) was conventionally ventilated and was ventilated according to OLC in a randomized cross-over setting. Conventional mechanical ventilation (CMV) consisted of volume-controlled ventilation with 5 cmH_2_O positive end-expiratory pressure and a tidal volume of 8–10 ml/kg. No recruitment maneuvers were performed. During OLC ventilation, recruitment maneuvers were applied until PaO_2_/FiO_2 _> 60 kPa. The peak inspiratory pressure was set to obtain a tidal volume of 6–8 ml/kg. The cardiac output (CO), the RV preload, the contractility and the afterload were measured with a volumetric pulmonary artery catheter. A high-resolution computed tomography scan measured the whole lung density and left ventricular (LV) volumes.

**Results:**

The RV end-systolic pressure–volume relationship, representing RV afterload, during steady-state OLC ventilation (2.7 ± 1.2 mmHg/ml) was not significantly different compared with CMV (3.6 ± 2.5 mmHg/ml). Pulmonary vascular resistance (OLC, 137 ± 49 dynes/s/cm^5 ^versus CMV, 130 ± 34 dynes/s/cm^5^) was comparable between groups. OLC led to a significantly lower amount of atelectasis (13 ± 2% of the lung area) compared with CMV (52 ± 3% of the lung area). Atelectasis was not correlated with pulmonary vascular resistance or end-systolic pressure–volume relationship.

The LV contractility and afterload during OLC was not significantly different compared with CMV. Compared with baseline, the LV end-diastolic volume (66 ± 4 ml) decreased significantly during OLC (56 ± 5 ml) ventilation and not during CMV (61 ± 3 ml). Also, CO was significantly lower during OLC ventilation (OLC, 4.1 ± 0.3 l/minute versus CMV, 4.9 ± 0.3 l/minute).

**Conclusion:**

In this experimental study, OLC resulted in significantly improved lung aeration. Despite the use of elevated airway pressures, no evidence was found for a negative effect of OLC on RV afterload or LV afterload, which might be associated with a loss of hypoxic pulmonary vasoconstriction due to alveolar recruitment. The reductions in the CO and in the mean pulmonary artery pressure were consequences of a reduced preload.

## Introduction

The open lung concept (OLC) is a ventilation strategy intended to avoid atelectasis causing shear forces during repeated opening and closing of atelectatic lung areas [1,2]. This is achieved with a recruitment maneuver and an application of sufficient positive end-expiratory pressure (PEEP) to counterbalance retractive forces. This strategy increases intrathoracic pressure, however, which could increase the right ventricular (RV) afterload [3-7] and could reduce safety.

Many studies (without recruiting the lung) show that elevated airway pressures increase the RV afterload in patients with respiratory failure [3,6-8]. One reason for this increase in RV afterload is alveolar overdistention of aerated lung areas in the presence of atelectasis; another reason is the occurrence of hypoxic vasoconstriction in atelectatic lung areas, as shown in experimental studies by Duggan and colleagues [9] and Cramer and colleagues [10]. We have shown that avoiding atelectasis by application of OLC ventilation did not lead to an increased RV afterload in cardiac surgery patients, despite the use of increased airway pressures [11,12]. Data on RV afterload in these latter studies were obtained by means of a pulmonary artery catheter or use of echocardiography. These methods are often used for measuring RV afterload, but they have not yet been validated. In addition, in these latter studies we were not able to assess atelectasis and therefore could not demonstrate a relationship between RV afterload and atelectasis.

We therefore designed an experimental study, investigating RV afterload during OLC ventilation compared with a low airway pressure ventilation strategy allowing atelectasis. RV afterload is assessed by the load-independent [13] afterload marker end-systolic pressure–volume relationship (ESPVR) [14-16]. The amount of atelectasis was assessed with a multi-slice whole lung computed tomography (CT) scan. As the influence of OLC during steady-state ventilation on left ventricular (LV) afterload is unknown, LV volumes were also measured during the whole cardiac cycle using this multi-slice CT scan.

We hypothesized that when atelectasis is minimized by OLC ventilation, the RV afterload and LV afterload are not affected despite the use of higher mean airway pressures in an experimental lung injury model.

## Methods

The study was approved by the institutional animal investigation committee, and the care and handling of the animals were in accordance with the European Community guidelines. In 10 pigs weighing 32 ± 1.3 kg, anesthesia was induced with ketamine hydrochloride (35 mg/kg, intramuscularly) and midazolam (0.5 mg/kg, intramuscularly). The animals were tracheotomized, connected to a Servo ventilator 300 (Siemens-Elema, Solna, Sweden) and were ventilated in a volume-controlled mode, with pure oxygen, at a rate of 20 breaths/minute, a tidal volume of 8 ml/kg, a PEEP of 5 cmH_2_O and an inspiratory/expiratory ratio of 1:2. Neuromuscular block was induced with pancuronium bromide (0.5 mg/kg intravenously), and anesthesia was maintained with a continuous infusion of fentanyl (20 μg/kg/hour), midazolam (0.3 mg/kg/hour) and pancuronium bromide (0.3 mg/kg/hour).

After induction, an indwelling ParaTrend 7+ blood gas analyzer probe (Philips, Boblingen, Germany) was inserted in the carotid artery for continuous blood gas analyses. An 8-Ch Foley catheter was inserted into the femoral vein. A correct position in the inferior caval vein was assured by CT scan of the abdomen. To reduce the cardiac preload, the Foley balloon was inflated with 5 ml water. One CCO 774HF75 series pulmonary artery catheter (Edwards, Irvine, CA, USA) was inserted through the right internal jugular vein with the tip in the pulmonary artery (measuring pulmonary artery pressures), and another catheter was also inserted through the jugular vein with the tip in the right ventricle (measuring RV pressures).

Hemodynamic measurements consisted of the right atrial pressure, the right ventricular pressure, the pulmonary arterial pressure, and the pulmonary capillary wedge pressure (PCWP). The cardiac output (CO), the RV end-diastolic volume (REDV) and the RV ejection fraction were calculated using a Vigilence cardiac output computer (Edwards), connected with the pulmonary artery catheter. From these values, the pulmonary vascular resistance (PVR = (mean pulmonary artery pressure - PCWP)/CO × 79.9) and the RV end-systolic volume were calculated.

The ESPVR was considered in each animal. During each ventilation strategy, the ESPVR was measured by calculating the slope of the end-systolic pressure and volume obtained with and without inflation of the balloon on the Foley catheter in the inferior caval vein. RV stroke work was calculated by the following equation: 0.0136 × (mean pulmonary artery pressure - right atrial pressure) × stroke volume [14]. The preload recruitable stroke work was considered in each animal during each ventilation strategy as the slope of RV stroke work and REDV obtained with and without inflation of the balloon on the Foley catheter in the inferior caval vein. Systemic vascular resistance was calculated as: (mean arterial pressure – right atrial pressure)/CO × 79.9.

After instrumentation, respiratory failure was induced by repeated saline lavage (50 ml/kg; 37°C) as described by Lachmann and colleagues [17]. Lavages were repeated at 3-minute intervals until the PaO_2 _was below 13 kPa.

To minimize the effect of confounding variables, conventional ventilation and OLC ventilation were applied in a cross-over design. The order of the applied ventilation strategies was randomized by sealed envelopes. Ten minutes after the last lung lavage, the first ventilation strategy was started. Before each ventilation strategy, the ventilation was disconnected for 15 seconds, which has been shown to result in an immediate lung collapse [18] and was substantiated by the CT measurements. Conventional mechanical ventilation (CMV) was started with volume control ventilation at the following settings: tidal volume, 8–10 ml/kg; PEEP, 5 cmH_2_O; inspiratory/expiratory ratio, 1:2; FiO_2_, 1.0; and respiratory rate adjusted to achieve PaCO_2_, 4.5–5.5 kPa.

Ventilation according to the OLC was started by switching the ventilator to a pressure-controlled mode with a respiratory frequency of 40/minute. The FiO_2 _was set at 1.0, the PEEP was 10 cmH_2_O, the inspiratory/expiratory ratio was 1:1, and a driving pressure suitable to obtain a tidal volume of 6–8 ml/kg aiming at a PaCO_2 _of 4.5 and 5.5 kPa was used. A lung recruitment maneuver was performed by increasing the peak inspiratory pressure to 40 cmH_2_O during 10 seconds in order to increase the PaO_2_/FiO_2 _ratio to a value greater than 60 kPa. If this value was not reached, a recruitment maneuver was repeated by adding 5 cmH_2_O to the previous peak inspiratory airway pressure, up to a maximum peak inspiratory airway pressure of 60 cmH_2_O. If the PaO_2_/FiO_2 _ratio decreased slowly below 60 kPa after recruitment, indicating renewed lung collapse, the PEEP was increased with 2 cmH_2_O and the recruitment maneuver (again beginning at 40 cmH_2_O) was repeated. If the PaO_2_/FiO_2 _ratio decreased below 60 kPa during the study period, the PEEP was not increased but a new recruitment maneuver was performed.

All measurements were performed once before lung lavage (at baseline) and twice after lung lavage during each ventilation strategy. Following lung lavage, one CT scan of the thorax was made to confirm lung collapse. During both ventilation strategies, measurements were performed once without balloon inflation of the Foley catheter in the inferior caval vein and once with inflation (5 ml saline) of the balloon of the Foley catheter.

Fluid management during the study was based on the REDV provided by the pulmonary artery catheter. The REDV before lung lavage was considered the optimal REDV. After lung lavage (and a REDV below the optimal value), the REDV was treated with starch colloids (Voluven^®^, Fresenius Kabi, Bad Homburg, Germany). A decrease of REDV during inflation of the Foley balloon was not treated.

The CT-scan protocol was performed using a state-of-the-art 64-slice Sensation 64 CT scanner (Siemens Medical Solutions, Forchheim, Germany) with a 0.4 mm voxel size and a 330 ms gantry rotation time. Each scan was performed twice: first with a standard protocol for thoracic imaging (standard scan), and then with dedicated software able to synchronize the reconstructed image with the cardiac phase (electrocardiogram (ECG) gated scan) [19]. The scan parameters were as follows: number of slices, 64/rotation; individual detector width, 0.6 mm; effective spatial resolution, 0.4 mm^3^; 120 kV, 120 mA/s (900 mA/s for the ECG gated scan); feed:rotation, 58 mm/pitch:1 (11.52 mm/pitch:0.2 for the ECG gated scan); effective reconstructed slice thickness, 0.6 mm; and reconstruction increment, 0.4 mm. The standard scan was reconstructed as a volumetric dataset, and a slice was selected every 20 mm starting at the apex of the thorax for the analysis. For the assessment of the left ventricle a short-axis multiphasic reconstruction was performed, dividing the cardiac cycle (using as two R waves reference points) into 10 phases and the left ventricle into eight levels [20]. The standard thoracic scan was used to analyze the lung parenchyma by means of dedicated PulmoCT software (Siemens Medical Solutions). The ECG gated scan and the left ventricle were analyzed with a dedicated ARGUS software platform (Siemens Medical Solutions).

CT data analysis was performed in all cases by an experienced radiologist. For the lung parenchyma evaluation we used three main ranges of attenuation, measured in Houndsfield Units (HU), representing the usual location of tissues in the HU spectrum: -1000 HU to -600 HU as good aerated lung tissue (voxels with a prevalent content of air); -600 HU to -200 HU as poorly aerated lung tissue (mostly voxels with air and with some soft tissues or fluid); and -200 HU to +200 HU as nonaerated lung tissue (mostly voxels with a mixture of fat, fluid and soft tissues).

The operator segmented in a semi-automatic mode the lung parenchyma of the right lung slice by slice (usually between 10 and 14 slices depending on the phase of the experiment and on the size of the animal's lung). The results were expressed as percentages of each subrange of attenuation as compared with the total lung area.

For evaluation of the left ventricle, the endocardial contours were semi-automatically detected by the operator on the images reconstructed on the short axis. The eight levels throughout the left ventricle allowed a volumetric interpolation of the whole left myocardium, allowing calculation of the end-systolic volume and of the end-diastolic volume.

### Statistics

Between-group differences for hemodynamic parameters were tested with a paired, two-sided Student *t *test. Results are presented as the mean ± standard error of the mean. A relationship between the end-systolic pressure and volume was calculated for each pig and these regression coefficients were then averaged. The relationship between RV afterload and lung aeration was calculated by the Pearson's correlation coefficient.

## Results

Hemodynamic data are presented in Table 1. In summary, the mean pulmonary artery pressure, the CO, and the mean arterial pressure were higher during CMV compared with OLC ventilation.

**Figure 1:**
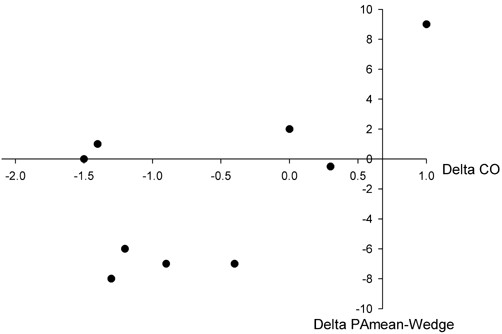
**Dynamic pressure-flow plot**. The effect of open lung ventilation (OLC) on flow and pressure drop through the pulmonary circulation is displayed compared with conventional mechanical ventilation (CMV). On the vertical axis, change of pressure drop through the pulmonary circulation is displayed: mean pulmonary artery pressure (PAmean)–pulmonary capillary wedge pressure during OLC – PAmean–pulmonary capillary wedge pressure during CMV. On the horizontal axis, the change of cardiac output (CO) is displayed.

As indicators of RV afterload, the regression coefficients between systolic pulmonary pressure and RV end-systolic volume were comparable between the two ventilation strategies (Table 1). Within the applied fluid management the dynamic pressure–flow diagram (Figure 1) showed a significantly lower CO during OLC (Table 1), but the pressure drop through the pulmonary circulation (pulmonary artery mean pressure - PCWP pressure) was not significantly higher during OLC ventilation (OLC, 6.0 ± 2.3 mmHg versus CMV, 7.4 ± 2.5 mmHg). The PVR was comparable between the two groups (Table 1).

**Figure 2:**
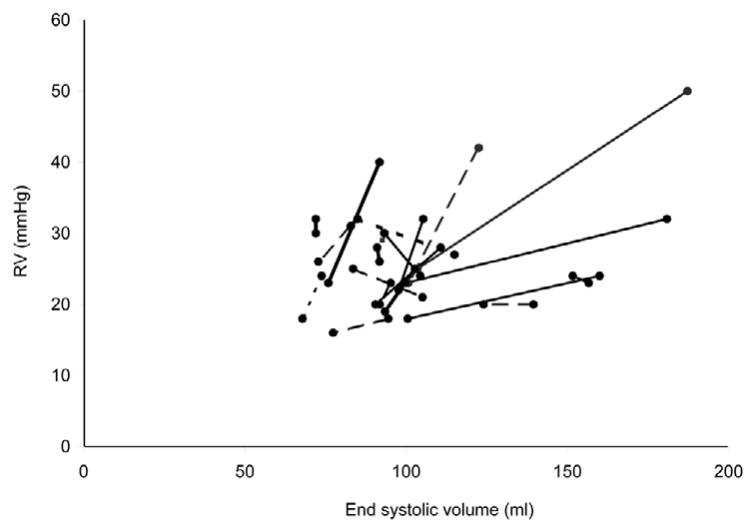
**End-systolic pressure–volume relationship**. The right ventricular end-systolic pressure (RV) versus the right ventricular end-systolic volume. The end-systolic pressure, and the volume with and without balloon inflation, is connected with a straight line for conventional mechanical ventilation and with the interrupted line for open lung ventilation.

Contractility in the right ventricle during OLC was not significantly different compared with during CMV. The regression coefficient of the ESPVR was comparable between the groups (OLC, 2.7 ± 1.2 mmHg/ml versus CMV, 3.6 ± 2.5; Figure 2). The regression coefficient of the preload recruitable stroke work was also no different between groups (OLC, 0.07 ± 0.07 g.m/beat.m^2^.ml versus CMV, 0.24 ± 0.16 g.m/beat.m^2^.ml; *P *= 0.36). The RV ejection fraction was also no different between the two groups (Table 1).

Contractility in the left ventricle during OLC was not significantly different compared with during CMV. The regression coefficient of the ESPVR was comparable between the groups (OLC, 43 ± 26 mmHg/ml versus CMV, 61 ± 30 mmHg/ml). The LV ejection fraction was also no different between the two groups (Table 1). The systemic vascular resistance, reflecting the LV afterload, tended to be lower during OLC ventilation compared with during CMV (*P *= 0.056) (Table 1).

**Figure 3:**
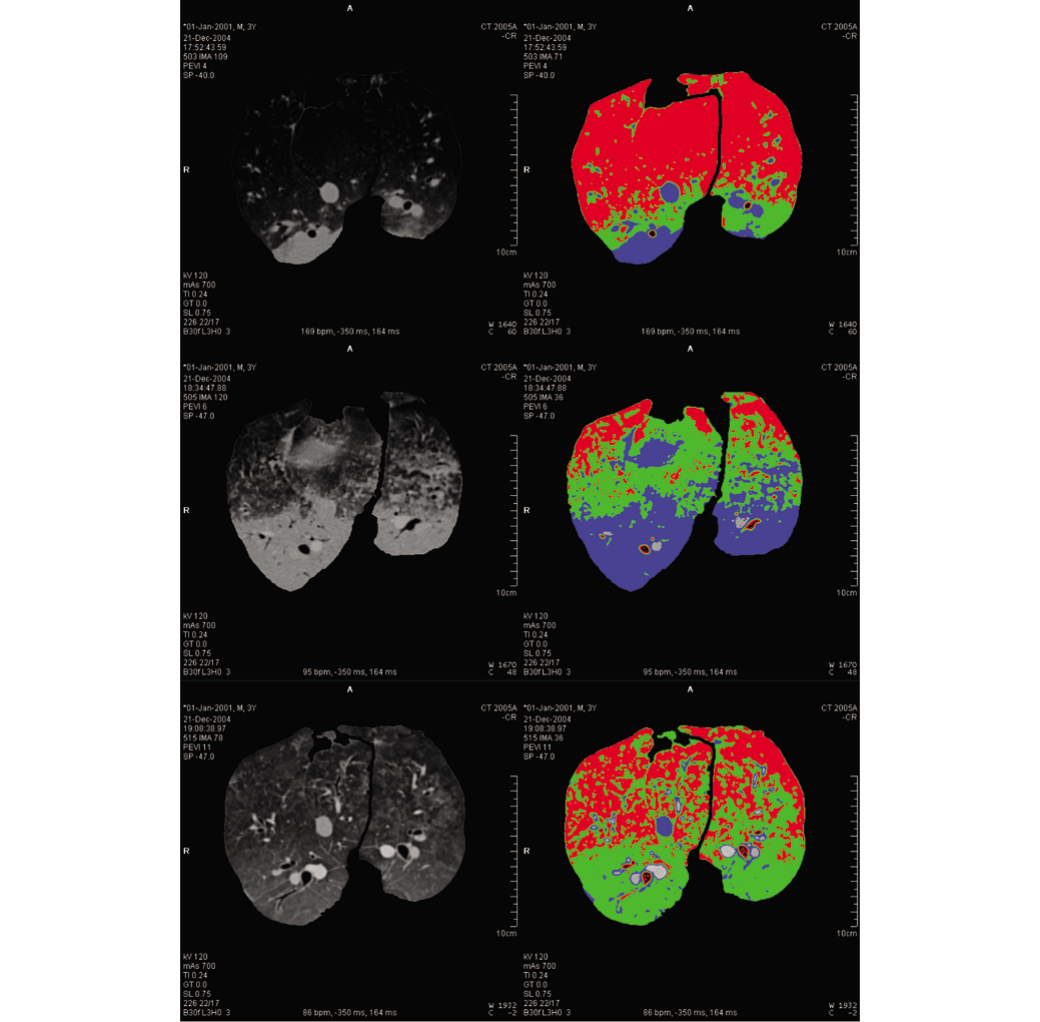
**Computed tomography scan examples of basal lung areasduring expiration**. Upper two scans, during baseline, before lung lavage. Middle two scans, conventional mechanical ventilation after lung lavage. Lower two scans, during open lung ventilation after lung lavage. Good aerated lung areas (-1000 Houndsfield units (HU) to -600 HU) are coded red in the right-hand scans, poorly aerated areas (-600 HU to -200 HU) are coded green, and non-aerated lung areas (-200 HU to +200 HU) are coded blue.

Considering the aeration of the lungs (Figure 3), 13 ± 2% of the lung was atelectatic during OLC whereas significantly more lung tissue was atelectatic in the CMV group (52 ± 3%, with a HU density between -200 and +200) (Table 2). The amount of poorly aerated lung tissue (HU density -600 to -200) was also significantly higher in the OLC group compared with the CMV group (Table 2). The amount of good aerated lung tissue (HU -1000 to -600) was also higher in the OLC group compared with the CMV group (Table 2). OLC ventilation could not, however, restore the area of good aerated lung tissue to baseline values (Table 2).

There was no significant correlation between the PVR, the CO and the pressure drop through the pulmonary circulation with the amount of lung aeration (Table 3).

## Discussion

In this experimental study, the amount of atelectatic lung area was not correlated with the parameters of RV afterload. OLC ventilation significantly increased the PaO_2_/FiO_2 _ratio and significantly reduced atelectasis compared with CMV. Indicators of RV afterload or contractility were not affected by the chosen ventilation strategy. Indicators of LV afterload and contractility were also no different between the different ventilation strategies.

This study showed that ventilation according to the OLC effectively reduced atelectasis. These findings are in agreement with results of Tusman and colleagues [21] and Amato and colleagues [22], who found that atelectasis is greatly reduced during OLC ventilation in children and in patients suffering from acute respiratory distress syndrome patients. The present study, however, also shows that there is still a small portion of nonaerated lung tissue during OLC ventilation. This is probably explained by the impossibility to exclude all (small) lung vasculature from lung density measurements. This falsely increases the amount of nonaerated lung tissue since lung vasculature has the same density as nonaerated lung tissue. This effect could be pronounced with this very high-resolution CT technique, also measuring very small pulmonary vessels. We therefore think that the amount of nonaerated lung tissue is negligible when considering the effect of OLC ventilation on RV contractility and RV afterload.

It is unlikely that OLC ventilation caused alveolar overdistention. The lung was less aerated during OLC compared with baseline. In some studies [23-25] overdistention (or emphysema) is characterized by the HU density ranging from -1000 HU to -900 HU. The limit between air and tissue in the lungs is arbitrary, however, because the spatial resolution dramatically affects the capability of the scanner to distinguish a voxel with air from a fluid/solid voxel on axial slices. Even with very high spatial resolution, as is the case in the present study (0.4 mm^3 ^is the highest available resolution for volumetric CT scanning), the distal part of the airways are too thin for this imaging modality. The borders of aerated lungs have recently been described as lower than -500 HU [25], while the limit for soft tissues is higher than -380 HU [26]. We therefore decided to have three homogeneous ranges of 400 HU, each starting at -1000 HU and ending at +200 HU.

In the present study, ESPVR, indicating RV afterload, was not correlated with atelectasis. This relationship was described by Duggan and colleagues [9] and Creamer and colleagues [10], who showed experimentally that atelectasis causes a significant increase in RV afterload. This effect of atelectasis on RV afterload during mechanical ventilation could be explained by two mechanisms: overdistention in aerated lung areas [27,28], and local hypoxic pulmonary vasoconstriction in nonaerated lung areas [29]. In the present study, we found no correlation between atelectasis and indicators of RV afterload. The effect of avoiding atelectasis (and thereby reducing hypoxic pulmonary vasoconstriction) by means of OLC ventilation on RV afterload is probably counterbalanced by the effect of a high intrathoracic pressure.

The RV afterload was not increased by the application of OLC ventilation. The mean arterial pulmonary pressure was even significantly decreased during OLC ventilation, suggesting a decreased RV afterload. This was not, however, consistent with other parameters of RV afterload. This decreased pulmonary artery mean pressure might be explained by a decreased preload during OLC ventilation. During OLC ventilation the CO decreased together with a decreased LV end diastolic volume, indicating a decreased preload. ESPVR is a load-independent afterload marker, and did not suggest a decreased RV afterload during OLC ventilation. We therefore think it is more prudent to state that RV afterload is unchanged during OLC ventilation.

The PVR is one of the parameters that indicate the RV afterload is unchanged during OLC ventilation. Using the PVR as an indicator of RV afterload, however, is heavily criticized [30]. Naeije [31] therefore proposed using a pressure–flow diagram; on the vertical axis the pressure drop through the pulmonary circulation (pulmonary artery mean pressure – PCWP) is displayed, and on the horizontal axis CO is displayed. Changes in pulmonary artery mean pressure – PCWP and changes in CO (the latter is also preload and contractility dependent) are compared with baseline values, indicating pulmonary vasoconstriction or dilatation. Despite the reduction of CO during OLC ventilation, the pulmonary artery mean pressure – PCWP value did not change, suggesting that RV afterload was not changed during OLC ventilation.

Another parameter reflecting ventricular afterload was proposed by Pinsky using the ESPVR [14]. When afterload varies while contractility is unaltered, as shown by the ESPVR, then the end-systolic pressure and volume varies – but along the line described by the ESPVR. The end-systolic pressure and volume did not differ significantly between the two ventilation strategies. In the case that RV contractility is not changed, therefore, the RV afterload is not affected by OLC ventilation.

RV contractility was comparable between both ventilation strategies. Ventricular contractility was assessed by the slope of the ESPVR and by the slope of the preload recruitable stroke work [32-34]. Both parameters adequately reflect contractility [13,32-34] and seem generally to be considered preload independent [13-16]. In addition, the ESPVR even correlated with myocardial oxygen consumption [35]. The slopes of both parameters were comparable, indicating an unchanged RV contractility during OLC ventilation. As the RV contractility did not change, the parameters for RV afterload were not affected by RV contractility – we therefore conclude that the RV afterload was not increased by application of OLC.

OLC also did not affect LV contractility and did tend to decrease LV afterload. The ESPVR, representing LV contractility, was not influenced by the applied ventilation strategy. The systemic vascular resistance, representing LV afterload, even tended to decrease during OLC ventilation. The CO and subsequently the mean arterial pressure, however, did decrease during OLC ventilation. The CO is preload, contractility and afterload dependent [14]. Indicators of LV preload, LV end-diastolic volume, LV contractility and LV afterload did not change significantly during OLC ventilation compared with during CMV. The LV end-diastolic volume, however, was significantly lower during OLC ventilation compared with baseline, whereas the LV end-diastolic volume during CMV was comparable with baseline. We therefore assume that a decrease of CO during OLC ventilation is primarily attributable to a preload effect. This hypothesis is supported by Wise and colleagues [36] and Fellahi and colleagues [37], who found no change of LV contractility during PEEP increment in patients with normal LV function [37].

## Conclusion

In this experimental study, OLC resulted in significantly improved lung aeration. Despite the use of elevated airway pressures, no evidence was found for a negative effect of OLC on RV or LV afterload that might be associated with a loss of hypoxic pulmonary vasoconstriction due to alveolar recruitment. The reduction in the CO and mean pulmonary artery pressure were consequences of a reduced preload.

## Financial disclosure

This study was sponsored by a grant from Edwards Life sciences. The authors have no financial disclosure.

## Key messages

• 	OLC improves lung aeration.

• 	OLC does not increase RV afterload.

## Abbreviations

CMV = conventional mechanical ventilation; CO = cardiac output; CT = computed tomography; ECG = electrocardiogram; ESPVR = end-systolic pressure–volume relationship; FiO_2 _= Inspired oxygen fraction;HU = Houndsfield units; LV = left ventricular; OLC = open lung ventilation; PaO_2 _= partial arterial oxygen pressure; PCWP = pulmonary capillary wedge pressure; PEEP = positive end-expiratory pressure; PVR = pulmonary vascular resistance; REDV = right ventricular end-diastolic volume; RV = right ventricular.

## Competing interests

This study was supported by a grant from Edwards LifeSciences.

## Authors' contributions

DRM participated in the design of the study, data acquisition and preparing the manuscript. LK participated in data acquisition and preparing the manuscript, JJH participated in the design of the manuscript, data acquisition and revising the manuscript, JJMT helped in the statistical analysis and revising the manuscript. BL and AJJCB helped in the design of the study and revising the manuscript. FC and AP participated in the design of the study, data acquisition and revising the manuscript. DG participated in the design of the study, fund acquisition, data acquisition and revising the manuscript.

## Figures and Tables

**Table 1:** Hemodynamic data at baseline and during conventional ventilation and open lung ventilation

	Baseline	Conventional mechanical ventilation	Open lung ventilation
Heart rate (beats/min)	105 ± 5	86 ± 5^**^	94 ± 5
Mean arterial pressure (mmHg)	93 ± 4	104 ± 4^**^	80 ± 4^*,**^
Right atrial pressure (mmHg)	4.2 ± 1	5.9 ± 1^**^	8.1 ± 2^**^
Cardiac output (l/min)	5.3 ± 0.3	4.9 ± 0.3	4.1 ± 0.3^*,**^
Right ventricular end-diastolic volume (ml)	165 ± 11	173 ± 13	148 ± 13
Right ventricular end-systolic volume (ml)	112 ± 10	119 ± 11	103 ± 11
Systolic pulmonary pressure (mmHg)	30 ± 3	31 ± 3	28 ± 2
Mean pulmonary arterial pressure (mmHg)	17 ± 3	20 ± 2	17 ± 2^*^
Pulmonary capillary wedge pressure (mmHg)	9.3 ± 2	12.1 ± 3	12.5 ± 2^**^
Right ventricular ejection fraction (%)	33.1 ± 1.7	33.1 ± 1.7	31.1 ± 1.9
Pulmonary vascular resistance (dynes/s/cm^5^)	126 ± 38	130 ± 34	137 ± 49
Left ventricular end-diastolic volume (ml)	66 ± 4	61 ± 3	56 ± 5^**^
Systemic vascular resistance (dynes sec cm^5^)	1379 ± 120	1693 ± 139^**^	1508 ± 124
Left ventricular ejection fraction (ml)	49.5 ± 1.6	53.2 ± 2.1	43.2 ± 5.6

^*^P < 0.05 open lung ventilation versus conventional mechanical ventilation, ^**^P < 0.05 versus baseline.

**Table 2:** Ventilatory measurements at baseline and during conventional ventilation and open lung ventilation

	Baseline	Conventional mechanical ventilation	Open lung ventilation
Intrinsic + extrinsic positive end-expiratory pressure (cmH_2_O)	5 ± 0.4	6 ± 0.3	14 ± 0.6^*,**^
Peak inspiratory airway pressure (cmH_2_O)	20 ± 0.5	28 ± 1^**^	26 ± 0.4^**^
Tidal volume (ml)	271 ± 5	270 ± 6	240 ± 11^*,**^
PaO_2_/FiO_2 _(kPa)	60 ± 5	13 ± 2	72 ± 2^*^
-1000 HU to -600 HU (%)	51 ± 3	10 ± 2^**^	29 ± 3^*,**^
-600 HU to -200 HU (%)	29 ± 2	36 ± 2	57 ± 3^*,**^
-200 HU to +200 HU (%)	20 ± 2	52 ± 3^**^	13 ± 2^*^

HU, Houndsfield units, expressed as the percentage of the lung area. ^*^P < 0.05 versus conventional mechanical ventilation, ^**^P < 0.05 versus baseline.

**Table 3:** Correlation between lung aeration and indicators of right ventricular afterload

Correlation coefficient	Good aeration (-1000 HU to -600 HU)	Poor aeration (-600 HU to -200 HU)	Nonaeration (-200 HU to +200 HU)
Pulmonary vascular resistance	0.7	-0.1	-0.7
Cardiac output	-0.2	-0.2	-0.2
Mean pulmonary artery pressure–pulmonary capillary wedge pressure^a^	0.2	-0.1	-0.7

HU, Houndsfield units, expressed as the percentage of the lung area. None of the correlations was significant. ^a^Pressure drop through the pulmonary circulation.
